# Environmental and nutritional assessment of young children’s diets in Norway: comparing the current diet with national dietary guidelines and the EAT-*Lancet* reference diet

**DOI:** 10.1007/s00394-023-03243-4

**Published:** 2023-08-31

**Authors:** Ellen Cecilie Wright, Bob van Oort, Marie Michaelsen Bjøntegaard, Monica Hauger Carlsen, Lene Frost Andersen

**Affiliations:** 1 Department of Nutrition, University of Oslo, Oslo, Norway; 2 CICERO Center for International Climate Research, Oslo, Norway

**Keywords:** Sustainable diet, Young children, Nutritional adequacy, Environmental impact, Global warming potential, Food-based dietary guidelines

## Abstract

**Purpose:**

Introducing healthy and sustainable diets early in life can promote lifelong healthy dietary patterns with a low environmental impact. Therefore, we aimed to estimate the environmental and nutritional consequences of a dietary change for 2-year-old children in Norway towards healthier dietary patterns.

**Methods:**

Environmental impacts of the current habitual diet among 2-year-olds (*n* = 1413) were estimated for six impact categories and compared with scenario diets based on the Norwegian food-based dietary guidelines (FBDG) and the EAT-*Lancet* Commission reference diet. Last, we evaluated the nutritional adequacy of the diets against the Norwegian nutrition recommendations for children aged 2–5 years. The current diet was assessed by an FFQ.

**Results:**

Environmental impacts of the current habitual diet were up to two times higher than those of the scenario diets. Compared with the current diet, impacts from the FBDG scenario diet were reduced by 35% for water use and 18% for terrestrial acidification, whereas impacts from the EAT-*Lancet* scenario diet were reduced by 51% for water use, 57% for terrestrial acidification, 36% for global warming potential and 27% for freshwater eutrophication. Milk and dairy products were the main contributors to environmental impacts in both the current diet and the FBDG scenario diet. The scenario diets were nutritionally adequate and improved the dietary quality among Norwegian 2-year-olds.

**Conclusion:**

Compared to current diets among young children, more plant-based dietary patterns in line with national FBDG or the EAT-*Lancet* Commission reference diet can improve the nutritional adequacy of diets and simultaneously reduce environmental impacts.

**Supplementary Information:**

The online version contains supplementary material available at 10.1007/s00394-023-03243-4.

## Introduction

Current dietary patterns have a negative impact on people’s health and on the natural environment, globally as well as locally [1–3]. In the Nordic countries, unhealthy diets are a leading risk factor for poor health and contribute to negative environmental impacts domestically and abroad [3]. A widespread shift in dietary patterns towards healthy diets from sustainable food systems is considered necessary to prevent malnutrition and diet-related non-communicable diseases, as well as reducing environmental pressure from the food system [2, 4]. Moreover, evidence suggests that food preferences and dietary habits are already established in childhood [5–8]. Introduction of healthy and sustainable dietary patterns early in life could therefore build the foundation for lifelong healthy dietary patterns with a low impact on the environment. Thus, it is important with knowledge about the environmental sustainability of current diets among young children and possible areas for improvements.

Healthy and sustainable dietary patterns are often described as predominantly plant-based diets [2, 9–11]. To aid transformations of dietary patterns towards more sustainable healthy diets, the EAT-*Lancet* Commission on Healthy Diets from Sustainable Food Systems described a universal healthy and environmentally sustainable reference diet, including a diversity of plant-based foods and low amounts of animal-sourced foods, highly processed foods and added sugars [2]. The reference diet should be suitable for adults and children and enable local adaptations to dietary preferences and food cultures of different populations.

In Norway, the national food-based dietary guidelines (FBDG) describe a healthy eating pattern tailored to Norwegian food culture [12]. While the guidelines are primarily based on health concerns, a separate assessment found its focus on increasing vegetable intake and limiting red meat consumption aligned with an environmentally sustainable diet [13].

To date, most research on the environmental sustainability of diets has focused on dietary patterns of adult populations [14–23] with a few exceptions [24–27]. In a Dutch study, the greenhouse gas emissions and blue water use of current diets were assessed among children aged 1–8 years [26] and, in a study from Italy, the carbon and ecological (land use) footprints from diets of 8- to 10-year-old children were assessed [25]. Among adolescents in Sweden, Colombo et al. assessed the carbon footprint of current diets and various optimised dietary patterns [27]. These studies all found unique environmental impact patterns for children and adolescents compared with adults, including which food groups contributed most to the different impact categories, showing the value of investigating children’s diets separately [25–27].

The aim of the present study was to estimate the environmental impacts (global warming potential, freshwater and marine eutrophication, terrestrial acidification, water use and land use) of current habitual diets among 2-year-old children in Norway and compare with environmental impacts of healthy scenario diets based on the Norwegian FBDG and the EAT-*Lancet* Commission reference diet, adjusted to young children aged 2 years. Furthermore, we evaluated the nutritional adequacy of the scenario diets against the Norwegian nutrition recommendations for children aged 2–5 years.

## Methods

The environmental impacts and nutritional content were estimated for three dietary patterns for 2-year-old children in Norway: the current habitual diet, a scenario diet based on the Norwegian FBDG and a scenario diet based on the EAT-*Lancet* reference diet [2, 11]. The diets were adjusted to 5.3 MJ/day, which is the reference value for energy intake for children aged 2–5 years [28].

### Current diet among 2-year-olds

Food consumption data collected through a dietary survey from 2019 [29] represented the current habitual diet. This nationally representative survey invited 2996 mothers of 2-year-old children to fill in a food frequency questionnaire (FFQ) about the usual diet of their child. Additional background information included the child’s gender, height, weight and childcare attendance, as well as some parental lifestyle variables and educational level. The participation rate was 47% (1413 individuals) and covered 49% girls and 51% boys. The sample of children was considered representative of Norwegian 2-year-olds in terms of gender distribution, birth weight, height and weight at 2 years of age and maternal age; however, the parent’s educational level was higher than the general population in Norway. 94% of the children attended childcare, and the food consumed at childcare was included in the habitual intake as reported by the parents.

The FFQ included questions about the habitual intake of around 200 food items. The FFQ was based on a previously validated FFQ from 2007 [30] and updated to include new food products on the market between 2007 and 2019. More information can be found in the original study report [29]. The participants reported the foods as eaten, including multi-ingredient foods, composite dishes and heat-treated foods in prepared form. To allow for analyses per food group and comparison between diets, the multi-ingredient foods and composite dishes were broken down into raw ingredients. For example, the reported intakes of pancakes (a multi-ingredient food) or lasagne (a composite dish) were broken down to the share of raw ingredients. Heat-treated foods were calculated in raw form, considering weight change during preparation. Only bread, oatmeal porridge, and industrially produced ready-made toddler dinners were not broken down into ingredients.

For descriptive purposes, the foods consumed in the dietary survey were grouped into 15 food groups based on the food groups in the EAT-*Lancet* reference diet. A detailed list of raw and prepared food products included in each food group is listed in supplementary information Table S1.

### Scenario diet for 2-year-olds based on the Norwegian food-based dietary guidelines

The Norwegian FBDG, presented in Table 1, was developed for a general healthy population and presents healthy food choices and intake levels from broad food categories [12]. The quantified food amounts stated in the guidelines are based on the food intake of a normal, physically active adult, but could be used for children and adolescents by adjusting amounts and portion sizes according to the energy requirements of these younger age groups [31]. When planning a diet for a healthy adult population, the reference energy intake is 10 MJ/day, whereas for children aged 2–5 years the reference energy intake is 5.3 MJ/day [28].

**Table 1:** The Norwegian food-based dietary guidelines [12]

	Food-based dietary guidelines^a^	Quantification of the FBDG according to an energy intake of 5.3 MJ/day
1	Enjoy a varied diet with lots of vegetables, fruit and berries, whole-grain foods and fish, and limited amounts of processed meat, red meat, salt and sugar	
2	Maintain a good balance between the amount of energy you obtain through foods and beverages and the amount of energy you expend through physical activity	5.3 MJ
3	Eat at least five portions of vegetables, fruit and berries every day. Half should be vegetables. For adults, one portion equals 100 g Potatoes and legumes are not included in the five portions, but should be part of a varied diet Eat a handful of unsalted nuts every day, around 20 g for adults	375 g 10 g
4	Eat whole-grain foods every day, providing 70–90 g wholemeal flour or whole grains for adults	40 g whole grain
5	Eat fish two to three times a week. You can also use fish as a topping or spread. For adults, this equals 300–450 g per week. At least 200 g should be fatty fish.	159–239 g per week, incl. ≥106 g fatty fish^b^
6	Choose lean meat and lean meat products. Limit the amount of red meat and processed meat to <500 g per week for adults	<265 g red and processed meat per week^c^
7	Include low-fat dairy products as part of your daily diet	
8	Choose cooking oils, liquid margarine and soft margarine spreads instead of hard margarines and butter	
9	Choose foods that are low in salt and limit the use of salt when preparing food and at the table	
10	Avoid foods and drinks that are high in sugar	
11	Choose water as a thirst quencher	For young children, approx. 65 ml/kg bodyweight per day

^a^The amounts are based on the food intake of a normal, physically active adult, 10 MJ/day

^b^The amounts refer to cooked weight. With a weight change factor of 0.8 [32], the corresponding raw weight is 199–299 g of total fish and at least 133 g of fatty fish per week

^c^The amount refers to cooked weight. With a weight change factor of 0.7 [32], the corresponding raw weight is <379 g/week

To construct a scenario diet for 2-year-olds in line with the Norwegian FBDG, the quantities in the guidelines were downscaled, where relevant, to match the reference value for energy intake for 2-year-olds, as shown in Table 1. The choice of foods was guided by healthy choices according to the guidelines as well as habitual food intake reported in the Småbarnskost 3 study [29], to reflect food preferences and a diet that is culturally acceptable for this age group. Moreover, the included amount of each food group reflected a possible daily intake through normal meals in line with Norwegian food culture, i.e. breakfast, lunch, dinner and an evening meal. The scenario diet included foods in the form commonly found at retail.

Daily consumption of at least five portions of vegetables and fruit is recommended. The portion size is not quantified for children, although ‘a handful’ is suggested as appropriate. A study on vegetable intake among young children in Norway suggested that a recommended intake for young children could be approximately 75% of what is recommended for adults [33]. Hence, the same was applied in the present study, resulting in a recommended daily intake of 375 g of vegetables and fruit, including 188 g of vegetables and 187 g of fruit.

The Norwegian Directorate of Health suggests a daily intake of three portions of low-fat dairy products, including at least two portions of milk or yoghurt [34]. As the guidelines do not include recommendations for children specifically, the amounts included in the present FBDG scenario diet are based on the Danish FBDG, which recommends a daily intake of 250 g of milk or yoghurt and 10 g of cheese for children aged 2–5 years [35].

In the present study, some discretionary foods and beverages were included to make the diet more realistic, and the amount of such foods included in the scenario diet is based on the Danish FBDG which specifies that, for the youngest children, ≤4% of the total energy intake could come from discretionary foods and beverages [35].

More details of the foods included in the scenario diet are shown in supplementary information Table S2. It should be noted that a broad variety of combinations of foods other than our constructed scenario could be created to fulfil the Norwegian FBDG.

### Scenario diet for 2-year-olds based on the EAT*-Lancet* reference diet

The EAT-*Lancet* reference diet is quantified with target values and possible ranges, for an intake of 2500 kcal (10.5 MJ)/day (Table 2) [2]. To construct a scenario diet for young children in line with the EAT-*Lancet* reference diet, the target values and ranges in the reference diet were adjusted to a daily energy intake of 5.3 MJ. Then, specific foods within each food group were selected guided by healthy choices and habitual food intake [29], thereby representing culturally acceptable foods for this age group.

**Table 2:** The EAT-*Lancet* reference diet recommended intake per food group, with a total energy intake of 10.5 MJ/day [2] and adjusted to a total energy intake of 5.3 MJ/day

Food groups	EAT-*Lancet* reference diet, intake based on an energy intake of 10.5 MJ/d^a^ (possible ranges)	EAT-*Lancet* reference diet, intake based on an energy intake of 5.3 MJ/d (possible ranges)
Whole grains, dry (g)^b^	232	117 (0–60% of total energy)
Potatoes (g)	50 (0–100)	25 (0–51)
Vegetables (g)	300 (200–600)	151 (101–303)
Fruits (g)	200 (100–300)	101 (51–151)
Dairy products, whole-milk equivalents (g)	250 (0–500)	126 (0–252)
Beef and lamb, raw (g)	7 (0–14)	4 (0–7)
Pork, raw (g)	7 (0–14)	4 (0–7)
Poultry, raw (g)	29 (0–58)	15 (0–29)
Eggs (g)	13 (0–25)	7 (0–13)
Fish, raw (g)	28 (0–100)	14 (0–51)
Legumes, dry (g)	75 (0–100)	38 (0–51)
Nuts^c^ (g)	50 (0–75)	25 (0–38)
Added fats^d^ (g)	51.8 (20–91.8)	26.1 (10–46.3)
Added sugars (g)	31 (0–31)	16 (0–16)

^a^The EAT-*Lancet* reference diet is based on a daily intake of 2500 kcal, which is equal to 10.5 MJ

^b^Includes whole grains and whole grain flour eaten raw or as an ingredient in bread, porridge, etc

^c^Includes peanuts and tree nuts

^d^Includes palm oil, unsaturated vegetable oils, lard and tallow

Some modifications were made to the original EAT-*Lancet* reference diet to make comparison between the diets in this study easier. The present scenario diet included foods in the form commonly found at retail. To reflect a realistic food intake, the grain products in the EAT-*Lancet* scenario diet include a mix of raw whole grains and commonly eaten whole-grain products such as bread, porridge and pasta—with a total whole grain (dry) content close to the EAT-*Lancet* reference diet target value. Moreover, the EAT-*Lancet* reference diet includes ‘whole milk or derivative equivalents’, whereas in the present scenario diet milk and yoghurt were included as processed products to represent actual consumed foods based on a whole milk equivalent factor of 1.0 for both milk and yoghurt.

In the EAT-*Lancet* reference diet, peanuts are categorised together with legumes, but, as peanuts are traditionally categorised as nuts in Norway, peanuts were included with nuts in the present study. Furthermore, the EAT-*Lancet* reference diet includes a small amount of palm oil and lard or tallow. However, as these fat types are neither healthy sources of fat nor commonly eaten in Norway, these fats were excluded in the present scenario diet. Finally, the reference diet allows for some added sugar, and the same amount of discretionary foods and beverages were included in both the FBDG and the EAT-*Lancet* scenario diets, i.e. ≤4% of the total energy intake.

In the EAT-*Lancet* scenario diet constructed for this study, the target values for each food group were strictly followed. However, other combinations of foods in addition to this scenario could still be within the suggested ranges of the EAT-*Lancet* reference diet (Table 2). More details of the foods included in the scenario diet are shown in supplementary information Table S2.

### Estimates of environmental impacts from the diets

A database with environmental impact values based on life cycle assessment (LCA) data for food items has been compiled and incorporated into the food composition and food and nutrition calculation system KBS, database version AE-22, at the Department of Nutrition of the University of Oslo. The impact categories included in the database and the present study are global warming potential, freshwater eutrophication, marine eutrophication, terrestrial acidification, water use, and land use. These impact categories were chosen based on their importance in food systems and the availability of data in the literature. Most of the available environmental data were estimated with the assessment method ReCiPe 2016 [36].

The impact category values were compiled from published LCA studies. Systematic literature searches for LCA studies were applied to food products representing the Norwegian market, including domestic and imported produce. The life cycle stages examined in the database includes primary production, processing, packaging, distribution, retail, storage, preparation by consumer and all waste along the life cycle excluding retail and household waste. Transport between all phases was included, except from retail to household. Identified data gaps in the life cycle stages of foods and missing food products were filled by the authors by using proxy values from similar foods in SimaPro (version 9.0.0.4.9) using the Ecoinvent 3 or Agri-footprint 4 databases [37–40], or from a Dutch environmental impact database [41]. Most of the LCA studies identified in the literature searches presented data on single food items or raw commodities and not composite dishes, while in the KBS AE-22 database impact category values for composite dishes and cooked food items can be automatically calculated based on recipes, including impacts from home preparation where relevant. The system boundaries and the functional units applied in the original LCA studies from which the database is compiled were decisive for whether food waste was included in the final impact category values for different foods. Therefore, avoidable and unavoidable food loss has not been added if not included in the original LCA studies.

In the present study, the dietary environmental impacts did not include impacts from consumer preparation at home. This was done because the Norwegian FBDG and the EAT-*Lancet* reference diet refers to food at the raw products level. Thus, to be able to assign the environmental impacts to approximately the same level of food categories (e.g. vegetables, dairy, meat, etc.) as the Norwegian FBDG and the EAT-*Lancet* reference diet, the present study present food data at retail level. Hence, the system boundary applied in the present study included primary production to retail.

The analyses were performed in KBS database version AE-22 and Microsoft Office Excel. For the current diet, environmental impacts were calculated on an individual level for all participants of the dietary survey and descriptive statistics were used to describe the environmental impacts on group level, including measures of mean, standard deviation and first and third quartiles.

### Estimates of nutritional content of the diets

Estimates of micro- and macronutrient content of the diets were based on the food intake reported in the FFQ and performed in KBS database version AE-22 [42]. When assessing the nutritional adequacy of the three diets, the aim was to assess whether the diets were adequate for dietary planning for a group of 2-year-old children, rather than evaluating individual nutrient intakes. Hence, only the population average nutrient content of the current diet was estimated, in addition to the nutrient content of the constructed scenario diets, and then compared with the recommended intake (RI) for children aged 2–5 years [28]. RIs are expressed as average daily intakes over time and refer to the amount of a nutrient that meets the known requirements among healthy individuals in a specified age interval and gender [43].

### Sensitivity analysis of the EAT-*Lancet* scenario diet

The EAT-*Lancet* reference diet includes ranges for their recommended intake of all food groups. Strictly following the target values of the EAT-*Lancet* reference diet did not meet the recommended intake of calcium, selenium or iodine. We therefore constructed two additional scenarios to test whether the recommended intake of all nutrients, according to the Norwegian nutrition recommendations [28], could be met within the proposed ranges adjusted to a daily energy intake of 5.3 MJ. The first additional scenario diet included twice the amount of dairy products (252 g) compared to the target value (126 g), hereafter called the ‘EAT-*Lancet* dairy’ scenario diet. The second additional scenario diet included twice the amount of dairy products and twice the amount of fish (252 and 28 g, respectively), hereafter called the ‘EAT-*Lancet* dairy & fish’ scenario diet. The total energy of the two additional scenario diets was adjusted to 5.3 MJ by reducing the content of vegetable oils as the EAT-*Lancet* diet includes relatively high amounts of added fats and to minimise the risk of reduction in micronutrients from other more nutrient-rich foods.

## Results

### Food composition of the diets

Among 2-year-olds in Norway the mean energy intake was 5.3 MJ/day, which is similar to the reference value for energy intake for this age group. This allowed for a comparison between the current diet and the scenario diets without any energy adjustment of the current diet. The food compositions of the three diets are presented in Table 3, per food group. More details on the food content of the three diets can be found in supplementary information Table S2.

**Table 3:** Food composition per food group in the current diet among 2-year-olds in Norway (Småbarnskost 3, *n* = 1413) [29], the FBDG scenario diet, and the EAT-*Lancet* scenario diet^a^

	Current diet (g/day)	FBDG scenario diet (g/day)	EAT-*Lancet* scenario diet (g/day)
Grain products^b^	222	264	296
*Whole-grain*^c^	*62*	*92*	*115*
Potatoes	14	30	25
Vegetables	71	188	151
Fruit, incl. juice^d^	217	187	101
Legumes	6	15	38
Nuts	0	10	25
Added vegetable fats	12	15	20^e^
Dairy products	489	260	126
Red meat	31	25	8
Poultry	7	20	15
Eggs	12	10	7
Fish	32	40	14
Sweets^f^	89	30	30
Drinking water	475	780	780
Other^g^	29		

All diets correspond to a daily energy intake of 5.3 MJ

Italic text and values indicate sub-groups and sub-group values

^a^Most foods are in raw form; see supplementary information Tables S1 and S2 for more details

^b^Includes a mix of bread, porridge, dry pasta and rice, and raw grains

^c^This refers to the whole-grain content of the grain products

^d^The current diet includes 129 g fruit and berries and 88 g fruit smoothies and juice, while the FBDG and EAT-*Lancet* scenario diets include fruit and berries only

^e^Includes unsaturated vegetable oils only, and excludes palm oil, lard and tallow which is included in the reference diet

^f^Includes sweets, chocolate, desserts, sugar-sweetened beverages and artificially sweetened beverages

^g^Includes plant-based beverages, formula milk, industrially produced ready-made toddler dinners and added salt and condiments

Compared with the FBDG and EAT-*Lancet* scenario diets, the current diet included fewer grain products, potatoes, vegetables, legumes, nuts and poultry, and more fruit, dairy products and sweets. The EAT-*Lancet* scenario diet included substantially fewer animal-sourced foods, including dairy products, red meat and fish, and more plant-based foods such as legumes, nuts and added vegetable fats than the other two diets.

### Environmental impacts of the diets

The estimated daily environmental impacts of the average current diet among 2-year-olds in Norway are presented in Table 4.

**Table 4:** Total daily environmental impacts per impact category of the current diet among 2-year-olds in Norway (Småbarnskost 3, *n* = 1413) [29]: mean (SD), first and third quartiles (Q1 and Q3)

	Mean (SD)	Q1; Q3
Global warming potential (kg CO_2_-equiv.)	2.1 (0.6)	1.6; 2.4
Freshwater eutrophication (g P-equiv.)	0.5 (0.2)	0.4; 0.6
Marine eutrophication (g N-equiv.)	2.3 (0.8)	1.8; 2.8
Terrestrial acidification (g SO_2_-equiv.)	24.5 (8.4)	18.8; 29.0
Water use (m^3^)	0.4 (0.2)	0.3; 0.5
Land use (m^2^a crop-equiv.)	2.1 (0.6)	1.6; 2.4

The current diet included a daily energy intake of 5.3 MJ

**Fig. 1:**
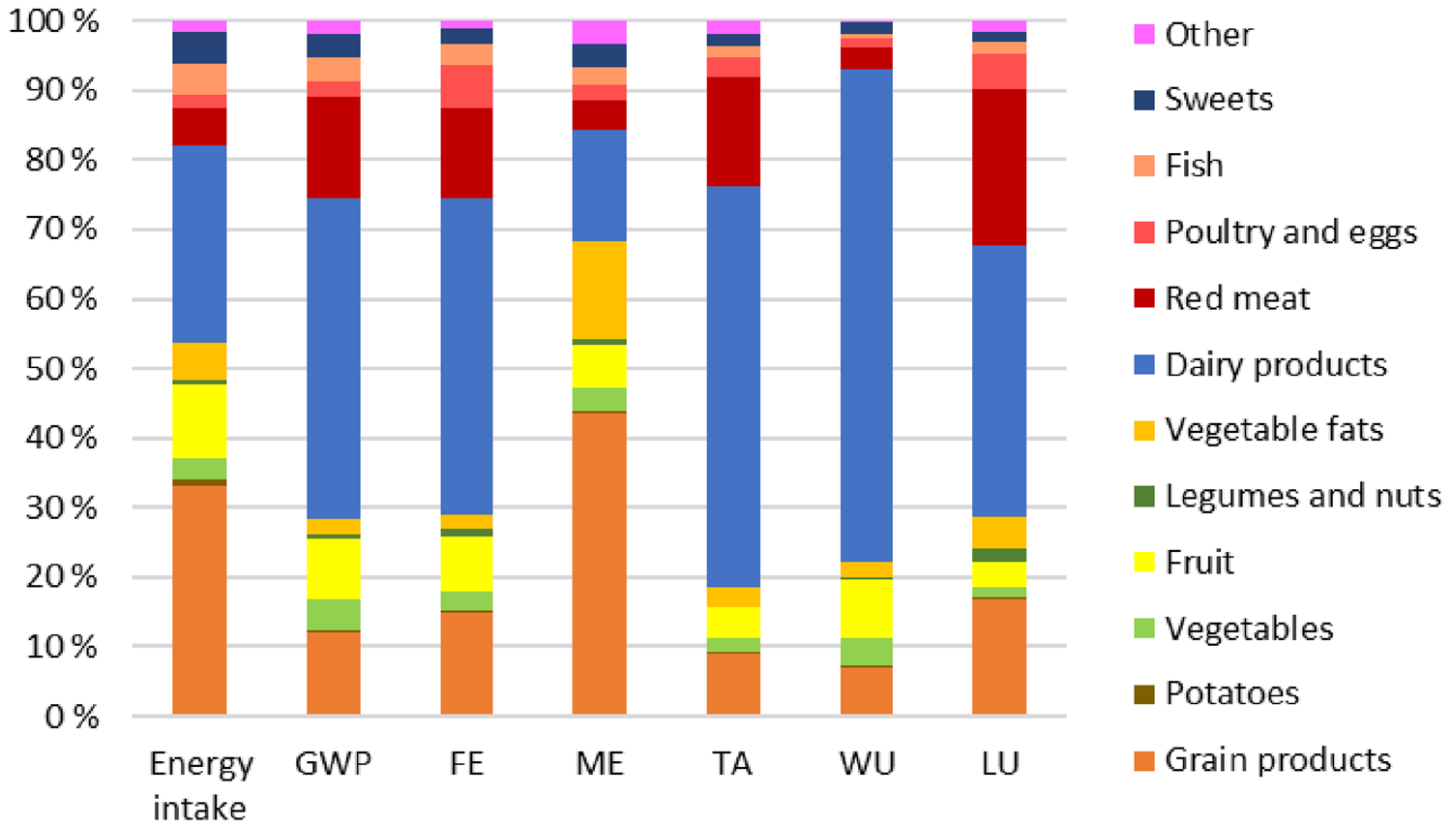
Relative contribution to the environmental impact categories from food groups in the current diet among 2-year-olds in Norway (Småbarnskost 3, *n* = 1413) [29]. ‘Sweets’ includes sweets, chocolate, desserts, sugar-sweetened beverages and artificially sweetened beverages. ‘Other’ includes drinking water, plant-based beverages, formula milk, industrially produced ready-made toddler dinners and added salt and condiments. *GWP* global warming potential; *FE* freshwater eutrophication; *ME* marine eutrophication; *TA* terrestrial acidification; *WU* water use; *LU* land use

The relative environmental impacts of different food groups in the current diet are shown in Fig. 1. Dairy products were the food group contributing most to environmental impacts, with 46% of the overall global warming potential, 46% of freshwater eutrophication, 16% of marine eutrophication, 57% of terrestrial acidification, 71% of water use and 39% of land use. Red meat contributed with 15% of global warming potential, 13% of freshwater eutrophication, 16% of terrestrial acidification and 23% of land use, and less to marine eutrophication (4%) and water use (3%). Grain products contributed most to marine eutrophication (44%) and between 7% and 17% to the other impact categories. In total, plant-based foods contributed with 54% of the energy intake and between 19% and 29% of the environmental impacts, except marine eutrophication where the contribution from plant-based foods was 68%. Animal-sourced foods contributed with 40% of the total energy intake and between 66% and 78% of the environmental impacts, except marine eutrophication where the contribution from animal-sourced foods was 25%. The remaining energy intake and environmental impacts were from sweets and other mixed foods.

**Fig. 2:**
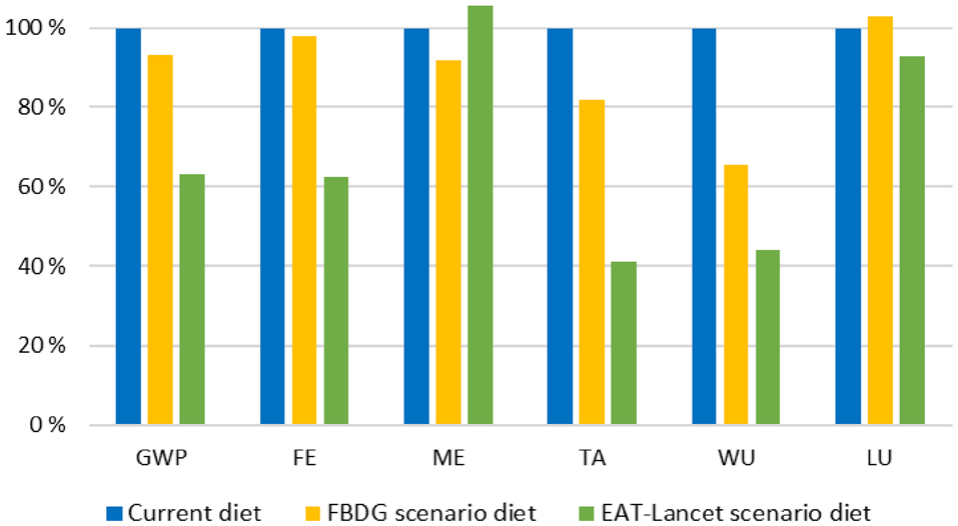
Total environmental impact per impact category from the FBDG scenario diet and the EAT-*Lancet* scenario diet in relation to the impacts from the current diet among 2-year-olds in Norway (Småbarnskost 3, *n* = 1413) [29]. *GWP* global warming potential; *FE* freshwater eutrophication; *ME* marine eutrophication; *TA* terrestrial acidification; *WU* water use; *LU* land use

In Fig. 2, the environmental impacts from the scenario diets are presented in relation to the current diet. Changing from the current diet to the FBDG scenario diet, the environmental impacts were reduced by 7% for global warming potential, 2% for freshwater eutrophication, 8% for marine eutrophication, 18% for terrestrial acidification and 35% for water use, and increased by 3% for land use. Changing from the current diet to the EAT-*Lancet* scenario diet, the environmental impacts were reduced by 37% for global warming potential, 38% for freshwater eutrophication, 59% for terrestrial acidification, 56% for water use and 7% for land use, and increased by 5% for marine eutrophication.

**Fig. 3:**
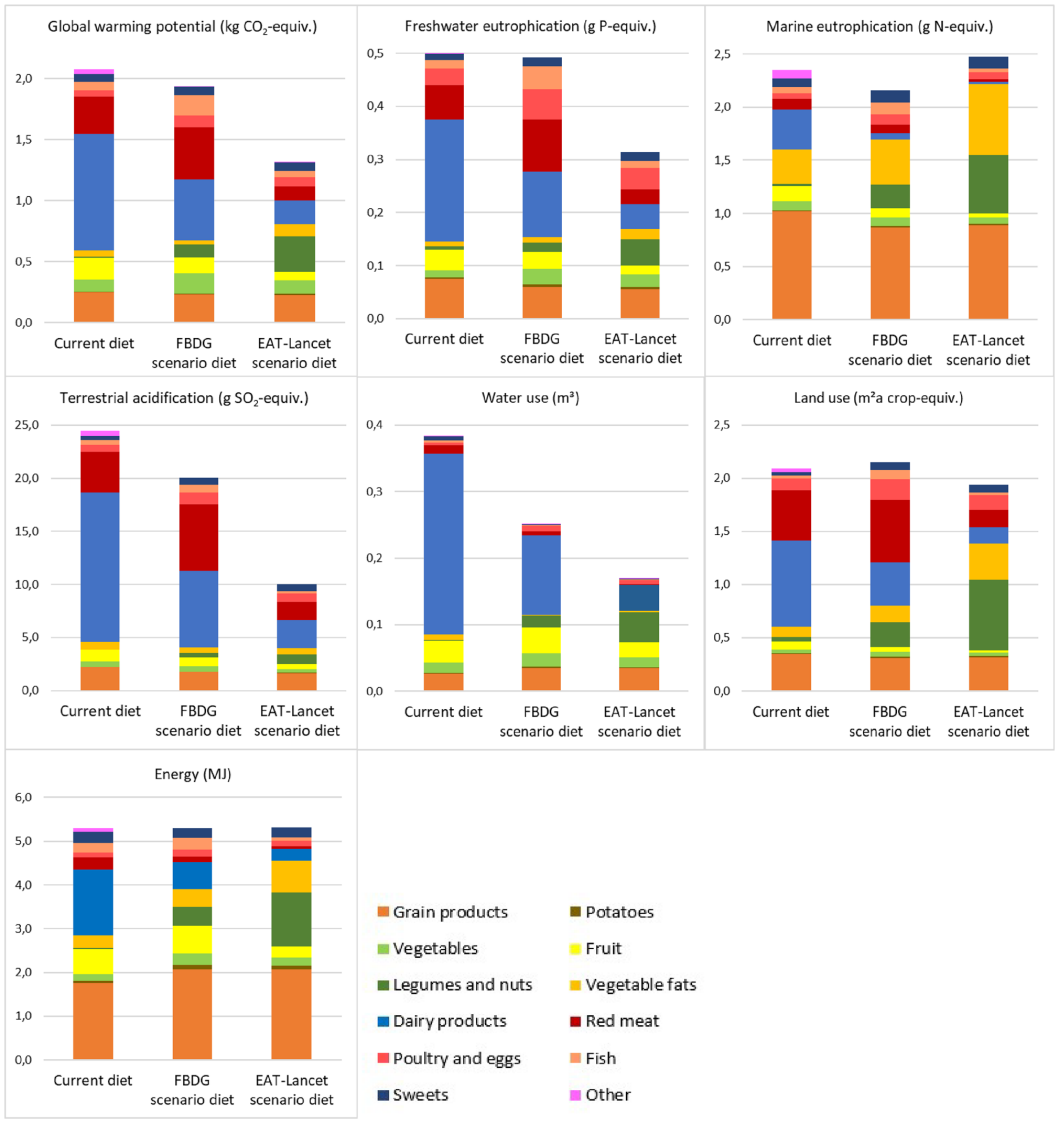
Environmental impacts from the current diet among 2-year-olds in Norway (Småbarnskost 3, *n* = 1413) [29], the FBDG scenario diet and the EAT-*Lancet* scenario diet, in total and per food group. ‘Sweets’ includes sweets, chocolate, desserts, sugar-sweetened beverages and artificially sweetened beverages. ‘Other’ includes drinking water, plant-based beverages, formula milk, industrially produced ready-made toddler dinners and added salt and condiments

Figure 3 shows the total environmental impacts per impact category for the three diets, as well as the proportional impact from each food group. Similar to the current diet, dairy products in the FBDG scenario diet contributed most to global warming potential, freshwater eutrophication, terrestrial acidification and water use, whereas marine eutrophication was largely affected by grain products. For land use, red meat contributed the most. In the EAT-*Lancet* scenario diet the contributions of the food groups to the different impact categories were more evenly distributed, although notable contributions from grain products, vegetable fats and legumes and nuts were observed for marine eutrophication and land use.

### Nutritional adequacy of the diets

In Table 5, the content of macro- and micronutrients in the current diet and the scenario diets was compared with the RI for nutrients according to the Norwegian nutrition recommendations [28].

**Table 5:** Content of macro- and micronutrients in the current diet among 2-year-olds in Norway (Småbarnskost 3, *n* = 1413) [29], the FBDG scenario diet, the EAT-*Lancet* scenario diet and the recommended intake for children aged 2–5 years according to the Norwegian nutrition recommendations [28]

	Current diet	FBDG scenario diet	EAT-*Lancet* scenario diet	Recommended intake
Energy (MJ)	5.3	5.3	5.3	5.3
Protein (E%)	17	19	16	10–20
Total fat (E%)	31	28	37	25–40
*Saturated fat (E%)*	***12***	*7*	*6*	*<10*
*Monounsaturated fat (E%)*	*12*	*12*	*17*	*10–20*
*Polyunsaturated fat (E%)*	*5*	*6*	*10*	*5–10*
Total carbohydrates (E%)	49	50	**43**	45–60
*Added sugar (E%)*	*4*	*2*	*2*	*<10*
Dietary fibre (E%)	3	4	4	
Dietary fibre (g/MJ)	3	5	5	2–3
Whole-grain (g/MJ)	12	17	22	>7.5
Vitamin A (µg)	804	549	426	350
Vitamin D (µg)	**4.5**	**4.1**	**1.3**	10
Vitamin E (mg)	7	9	14	5
Thiamine (mg)	1.1	1.3	1.4	0.6
Riboflavin (mg)	1.5	1.0	0.7	0.7
Niacin (mg)	**8**	13	11	9
Vitamin B_6_ (mg)	1.0	1.4	1.2	0.7
Folate (µg)	187	258	316	80
Vitamin B_12_ (µg)	5.3	3.6	1.7	0.8
Vitamin C (mg)	64	81	59	30
Calcium (mg)	942	640	**431**	600
Iron (mg)	**6.3**	8.4	10.3	8
Zinc (mg)	7.4	7.8	7.5	6
Selenium (µg)	31	28	**21**	25
Iodine (µg)	133	90	**55**	90
Salt (g)	3.9	2.2	1.6	<3–4

Italic text and values indicate sub-groups and sub-group values. Bold values indicate values that do not meet the recommended intake

In the current diet, intake of most macro- and micronutrients was within the RI, whereas the intake of saturated fat was above the RI, and the intake of vitamin D and iron was below the RI. However, the dietary shifts from the current diet to the scenario diets resulted in lower content of saturated fat and higher content of iron. In the FBDG scenario diet, the content of macro- and micronutrients met the RI for all nutrients, except for vitamin D. In the EAT-*Lancet* scenario diet, the content of carbohydrates was below the RI, as well as the content of vitamin D, calcium, selenium and iodine.

Table 6 presents the content of vitamin D, calcium, selenium and iodine in the three EAT-*Lancet* scenario diets. With double amounts of dairy products (the EAT-*Lancet* dairy scenario), calcium reached the RI, whereas the RI for selenium and iodine were reached with double amounts of dairy products *and* fish (the EAT-*Lancet* dairy and fish scenario). Vitamin D was below the RI for all scenario diets. More details about the additional EAT-*Lancet* scenario diets are presented in supplementary information Table S3.

**Table 6:** Content of vitamin D, calcium, selenium and iodine in the three different EAT-*Lancet* scenario diets, and the recommended intake for children aged 2–5 years according to the Norwegian nutrition recommendations [28]

	EAT-*Lancet* scenario diet	EAT-*Lancet* dairy	EAT-*Lancet* dairy and fish	Recommended intake
Vitamin D (µg)	**1.3**	**1.6**	**2.4**	10
Calcium (mg)	**431**	608	610	600
Selenium (µg)	**21**	**23**	27	25
Iodine (µg)	**55**	**75**	98	90

Bold values indicate values that do not meet the recommended intake

When the amounts of dairy products and fish were increased in the additional EAT-*Lancet* scenario diets, the global warming potential, terrestrial acidification and water use increased compared with the EAT-*Lancet* scenario diet based on the target values, whereas marine eutrophication decreased and freshwater eutrophication and land use remained similar (supplementary information Fig. S1).

## Discussion

To the authors’ knowledge, this was the first study to assess the environmental impact across six impact categories from diets among 2-year-old children. We observed that in the current diet the intake of dairy products was the most important contributor to five out of six environmental impact categories, followed by red meat and grain consumption. Furthermore, the environmental impacts of the current diet were found to be higher than impacts from the modelled scenario diets based on the Norwegian FBDG and the EAT-*Lancet* reference diet. The highest reductions in environmental impacts were found when shifting from the current diet to the EAT-*Lancet* scenario diet. Last, we showed that nutritionally adequate scenario diets for young children could be constructed within the recommendations of the Norwegian FBDG and the intake ranges of the EAT-*Lancet* reference diet.

On average, the current diet of Norwegian 2-year-olds contributed with 2.1 kg CO_2_-equiv., 0.5 g P-equiv., 2.3 g N-equiv. and 24.5 g SO_2_-equiv. and used 0.4 m^3^ of water and 2.1 m^2^ a crop-equiv. of land per day. Most other studies that have assessed the environmental impact of diets have focused on global warming potential. Among older children in Italy and the Netherlands, the daily global warming potential per person was similar and slightly higher than observed in the present study: 2.2 and 3.0 kg CO_2_-equiv., respectively [25, 26]. Among adolescent and adult populations in Europe, the per person daily dietary global warming potential ranged between 3.4 and 6.0 kg CO_2_-equiv. [15, 20, 23, 26, 27]. The lower observed environmental impacts among the 2-year-olds compared with older populations were partly due to a lower energy intake. When adjusting for energy intake, the average global warming potential for Norwegian 2-year-olds was 0.4 kg CO_2_-equiv./MJ compared with 0.4 and 0.5 kg CO_2_-equiv./MJ among children in Italy and the Netherlands, respectively [25, 26], and between 0.4 and 0.6 kg CO_2_-equiv./MJ among adolescents and adults in Europe [15, 20, 23, 26].

The studies among older children in Italy and the Netherlands assessed land use and water use, respectively, in addition to global warming potential [25, 26]. Direct comparison between studies is however problematic due to common methodological differences between studies, such as the system boundary applied and LCA data source. The average water use among children in the Netherlands was found to be 0.1 m^3^ per day (compared to 0.4 m^3^ per day among Norwegian children) [26], while the method used to assess land use in the study from Italy was not comparable to the method used in the present study [25]. Further, the studies on environmental impacts of children’s diets in Italy and the Netherlands [25, 26] both included impacts from home preparation, which was excluded in the present study. However, when impacts from home preparation were included in the estimated environmental impacts from the current diet among 2-year-olds in Norway, the total impacts per impact category were less than 1% higher, indicating a very low contribution from home preparation to the total environmental impacts,

Similar to findings among other population groups, animal-sourced foods contributed the most to global warming potential of current diets among young children in Norway. However, intake of meat is often the most important contributor to global warming potential from adult diets [15, 20, 23, 26], while consumption of dairy products was the most important contributor from the diets among 2-year-olds in Norway. Dairy products contributed with 46% of global warming potential whereas meat (red meat and poultry combined) contributed with 16%. In European adult populations, the contributions to global warming potential from dairy products consumption were between 13% and 23% whereas the contributions from meat consumption were between 30% and 38% [15, 20, 23, 26]. Vellinga et al. similarly found that the contribution of dairy products to global warming potential was more prominent among young children (1–8 years) compared with older children and adults [26].

In the present study, the intake of dairy products was the most important contributor to the other impact categories as well, except marine eutrophication. For marine eutrophication, grain consumption was the largest contributor. The content of fruit and vegetables is commonly found to strongly contribute to the water use of diets [9, 26]. However, we found that, in the current diet among 2-year-olds in Norway, fruit and vegetables contributed with only 9% and 4%, respectively.

Changing from the current diet to a diet in line with the Norwegian FBDG reduced the overall environmental impact, whereas changing to a diet aligned with the EAT-*Lancet* reference diet reduced most of the environmental impacts even more. A notable dietary change from the current diet to the scenario diets was the inclusion of less dairy products. A high consumption of milk is a distinct characteristic of diets among young children compared with older children and adults [26, 28, 44–48]. Among Norwegian 2-year-olds, the average daily intake of milk and yoghurt was 460 g, and dairy products (in total 489 g including milk, yoghurt, cheese and butter) contributed with 28% of the total daily energy intake. The scenario diets included much less milk and yoghurt, i.e. 250 g in the FBDG scenario diet and 126 and 252 g in the EAT-*Lancet* scenario diets, as well as lower energy contribution from total dairy, 12% and 5%, respectively. Due to this large reduction of milk and dairy products content, the scenario diets were effective in reducing the environmental impacts of the diet especially for global warming potential, freshwater eutrophication, terrestrial acidification and water use. However, marine eutrophication was increased in the EAT-*Lancet* scenario compared to the other diets. In the EAT-*Lancet* scenario diet, the three main sources of energy were grains, vegetables oils and legumes and nuts—food groups that showed relatively high impacts on marine eutrophication, especially the vegetable oils. Further, land use was almost similar for all three diets, as the reductions in land use from dairy products in the EAT-*Lancet* scenario diet was outweighed by the increase in land use from vegetable oils, legumes and nuts.

Other potential reductions in environmental impacts of the current diet that could have been explored were to choose lower-impact foods within a food group [10, 41, 49–52]. For instance, there are large variations in the environmental impacts of meat, especially between red meat and poultry, but also between different types of red meat such as beef and pork [10, 41, 49]. Seafood is another food group in which the environmental impacts vary widely depending on the species, production and harvesting techniques, because in particular crustaceans and farmed fish have higher environmental impacts than many wild-caught fish species [10, 41, 49–52], although some wild fish species are under the pressure of over-fishing [2, 51].

Young children have unique nutritional needs to support rapid growth and development and require higher nutritional density in their diets than adults [53]. Plant-based diets generally result in lower environmental impacts compared with omnivorous dietary patterns [9, 27] and have been linked to a more favourable intake of many micronutrients and fatty acids among children aged 1–3 years [54]. However, if not carefully constructed, diets without animal-sourced foods may increase the risk of nutritional deficiencies among young children [54–56]. A study applying the EAT-*Lancet* reference diet to a Danish setting found that the dietary content of vitamin D, calcium, iron, iodine and zinc could be a concern among children [21]. In the present study we similarly observed challenges with reaching adequate levels of vitamin D, calcium, selenium and iodine in the EAT-*Lancet* scenario diet when strictly applying the target values of the EAT-*Lancet* reference diet. In the current diet, milk and other dairy products contributed with about 80%, 35% and 60% of the total calcium, selenium and iodine intake, respectively [29]. In addition, fish intake contributed with 23% and 17% of the selenium and iodine intake, respectively. The target values of the EAT-*Lancet* reference diet include a low amount of both dairy products and fish; however, when the amounts of these food items were increased within the recommended ranges to 252 and 28 g, respectively, the EAT-*Lancet* dietary scenario reached the RIs for calcium, selenium and iodine. The EAT-*Lancet* Commission did emphasise that some population groups, including young children, could benefit from higher consumption than the target values [2]. Our study demonstrates the importance of actively using the intake ranges proposed for the EAT-*Lancet* reference diet in addition to the target values, and not simply “downscale” according to energy intake from scenarios that have been created for adults. The FBDG scenario diet reached the RI for all nutrients (except vitamin D), including calcium, selenium and iodine.

The content of vitamin D was below the RI for children aged 2–5 years in all diets analysed in the present study. The challenge to reach adequate levels of vitamin D intake from the diet alone has been widely recognised, and Norway currently implements voluntary vitamin D fortification of low-fat milk, butter and margarine [57, 58]. Furthermore, the Norwegian health authorities recommend supplementation with vitamin D for infants from birth [59] and continued supplementation for children and adults with low intake of vitamin D-rich foods [57]. In the present study, intake of micronutrient supplements was not included in the analyses, whereas micronutrients fortified in foods were captured. The current diet included a mix of vitamin D-fortified and unfortified milk, according to the average food intake reported in the dietary survey. The scenario diets included vitamin D-fortified milk only. Inclusion of fortified plant-based beverages as a substitution for cow milk could potentially decrease the environmental impacts of the diets [10] while at the same time provide valuable micronutrients in diets with no or low cow milk intake. The scenarios in the present study did not include fortified plant-based beverages, and inclusion of such products in the EAT-*Lancet* scenario diet could have increased the content of e.g. calcium, selenium and iodine.

Additionally, we found that with the food composition of the scenario diets the dietary content of saturated fat was reduced and the dietary content of iron was increased compared with the current diet. This was perhaps due to a lower content of processed meat products, often high in saturated fat, and a higher content of lean meat, whole grain products and legumes contributing to the higher content of iron.

As dietary patterns seem to persist from early childhood into adolescence and adulthood [5–8], a preference for environmentally sustainable foods at an early age can lead to dietary choices with lower environmental impact later in life as well. This highlights the importance of establishing healthy and sustainable dietary patterns early in life. It can be argued that the role of the environmental impact of the diet of the youngest children is less important than among adults due to their lower energy intake. However, the present study has demonstrated that a dietary shift for young children from current diets towards diets aligned with the Norwegian FBDG and the EAT-*Lancet* reference diet can potentially reduce certain environmental impacts by up to 45% without compromising the nutritional adequacy. Hence, even in diets with a total energy intake of around 5 MJ/day, dietary shifts can contribute to overall reduced environmental pressure.

It should be noted that dietary change towards more environmentally sustainable diets is only one of several measures to reduce the environmental pressure from the food system. Other important measures that have not been addressed in the present study include improved food production practices and food waste reduction [2].

## Strengths and limitations

A strength of the present study was the simultaneous consideration of food-based dietary guidelines, nutrient adequacy and environmental impacts. This provides a more comprehensive picture, highlighting challenges of meeting adequacy on all dimensions at the same time.

Another strength of the present study was the comprehensive, updated and context-specific LCA database incorporated into the national food and nutrition calculation system, which allowed for automatic and simultaneous estimates of nutrient content and environmental impact of food items and diets. Moreover, it is a strength that we included estimation of six different environmental impact categories, because both similarities and specificities of the different impact categories were revealed.

A limitation of the study was that the scenario diets represented only a small fraction of possible diets in line with the Norwegian FBDG and the EAT-*Lancet* reference diet, which could result in both higher and lower environmental impacts and also better or worse nutritional quality. Compared with the Norwegian FBDG, the scenario diet in the present study was, in particular, on the lower side for meat and dairy products. The content of red meat and dairy products could have been almost doubled and still be within the recommendations of the guidelines. As these food groups represent high environmental impact foods, a dietary scenario with more red meat and dairy products—and less plant-based foods—would have been likely to result in higher environmental impacts.

Another limitation was the uncertainty in the environmental impact data from different LCA studies which applied different methodologies estimating the impact category values. Due to lack of data, avoidable and unavoidable food loss at retail and at home has not been considered at the present stage of the database.

As with all dietary assessment methods, there are limitations with FFQs. Portion sizes and frequency of consumption may be difficult to recall. Moreover, specifications of foods are limited due to the closed food list in the FFQ. The FFQ used to assess the current diet in the present study was based on an FFQ that was validated against 4-days weighed food records [30]. The validation indicated both over- and underestimation in intake of certain food groups and nutrients in the FFQ, although most of the food groups and nutrients did not show significant differences between the two methods.

## Conclusions

Transforming current diets among young children in Norway towards diets in line with the Norwegian FBDG or the EAT-*Lancet* reference diet could reduce the environmental impacts by up to around 50%. In the current diet, consumption of milk and other dairy products contributed the most to the environmental impacts, and the reduction in milk and dairy products content from the current diet to the scenario diets was driving the reduction in overall environmental impacts. Although young children may have specific nutritional needs, we found that nutritionally adequate diets could be constructed within the Norwegian FBDG as well as within the intake ranges of the EAT-*Lancet* reference diet.

If Norway or other countries wish to create healthy and environmentally sustainable FBDG for children in the future, the present study has exemplified some challenging topics such as the role of dairy in the diet. We have also shown that focusing on global warming potential as the only environmental impact factor may ignore stress on other environmental impact categories, such as marine eutrophication and water use.

### Supplementary Information

Below is the link to the electronic supplementary material.

Supplementary file1 (PDF 211 KB)

## Data Availability

The data used in the present study is not open access or publicly available, but the corresponding author can be contacted.
