# Supplementary material for: Environmental and nutritional assessment of young children’s diets in Norway: comparing the current diet with national dietary guidelines and the EAT-Lancet reference diet
Source: Eur J Nutr. 2023 Aug 31;62(8):3383–96. doi: 10.1007/s00394-023-03243-4 (PMC10611869; doi:10.1007/s00394-023-03243-4)
Supplement: Supplementary file 1 — Supplementary file1 (PDF 211 KB) [file 394_2023_3243_MOESM1_ESM.pdf]

# Supplementary Information

## Environmental and nutritional assessment of young children's diets in Norway: comparing the current diet with national dietary guidelines and the EAT-*Lancet* reference diet

*European Journal of Nutrition*

Ellen Cecilie Wright[*], Bob van Oort, Marie Michaelsen Bjøntegaard, Monica Hauger Carlsen, Lene Frost Andersen

[*] Corresponding author: Ellen Cecilie Wright; Department of Nutrition, University of Oslo, Norway; tel.: +47 99744145; e-mail: e.c.wright@medisin.uio.no

**Supplementary Table S1** Foods included in the food groups

| | | |
|---|---|---|
| **Grain products** | | Raw grains and breakfast cereals<br>Bread and porridge<br>Uncooked white and whole grain pasta and rice |
| **Potatoes** | | Raw potatoes |
| **Vegetables** | Dark green vegetables | Raw and boiled spinach and broccoli |
| | Red, orange vegetables | Raw and boiled carrots<br>Raw tomatoes and red pepper<br>Ketchup, tomato paste and crushed tomatoes |
| | Other vegetables | Raw cucumber, avocado, sweet corn, sugar snaps, cauliflower and onion |
| **Fruit, incl. juice** | Fruit and berries | Raw fruit and berries (mainly apple, banana, blueberries and orange)<br>Raisins |
| | Fruit smoothies | Fruit smoothies without added sugar |
| | Fruit juice | Fruit juice without added sugar |
| **Legumes** | | Dried, frozen or fresh beans, lentils and peas |
| **Nuts** | Nuts | Raw peanuts and tree nuts; peanut butter |
| **Added vegetable fats** | Vegetable oils | Mainly olive, rapeseed, sunflower and soy oil |
| | Margarine | Liquid and soft margarine based on vegetable oils |
| **Dairy products** | Milk | Whole, semi-skimmed and skimmed milk<br>Milk with flavour |
| | Yoghurt | Drinking yoghurt, yoghurt and "skyr" |
| | Cheese | Hard cheese, cheese spread and "brunost" |
| | Butter | Butter and butter mixes (Bremykt) |
| **Red meat** | Whole and minced red meat | Raw whole and minced meat of beef, pork and lamb |
| | Processed red meat products | Processed meat products with beef, pork or lamb (sausage, ham, salami, liver paste, etc.) |
| **Poultry** | Whole and minced poultry | Raw whole and minced meat of chicken and turkey |
| | Processed poultry products | Processed meat products with chicken or turkey (sausage, ham, liver paste, etc.) |
| **Eggs** | | Raw eggs |
| **Fish** | Lean fish | Raw fillets of cod and other white fish |
| | Fatty fish | Raw fillets of salmon and other fatty fish |
| | Processed fish products | Processed fish products (fish cakes, fish fingers, etc.) |

| | | |
|---|---|---|
| **Sweets** | Sweets, cakes and desserts | Jam, sweets, chocolate, cakes, sweet biscuits, ice-cream, etc. |
| | Sugar-sweetened beverages | Juice concentrate, soda and other sugar-sweetened drinks |
| | Artificially sweetened beverages | Juice concentrate, soda and other artificially sweetened drinks |
| **Drinking water** | | Tap water, mineral water |
| **Other** | Plant-based beverages | Mainly oat milk |
| | Formula milk | Mainly toddler formula prepared from powder |
| | Industrially produced toddler dinners | Ready-to-eat dinner meals including potatoes, vegetables, pasta, meat and/or fish |
| | Added salt and condiments | Salt, herbs, etc. |

**Supplementary Table S2** Food composition in the current diet among 2-year-olds in Norway (Småbarnskost 3, *n* = 1413) [1], the FBDG scenario diet and the EAT-*Lancet* scenario diet

| | **Småbarnskost 3** | | **FBDG scenario diet** | | **EAT-*Lancet* scenario diet** | |
|---|---|---|---|---|---|---|
| | Food (g/day) | Energy (kJ/day) | Food (g/day) | Energy (kJ/day) | Food (g/day) | Energy (kJ/day) |
| **Total** | **1729** | **5293** | **1874** | **5303** | **1639** | **5308** |
| **Grain products** | **222** | **1694** | **264** | **2073** | **295** | **2054** |
| Whole-grain bread[a] | 89 | 874 | 80 | 791 | 80 | 787 |
| White bread[b] | 31 | 294 | 35 | 352 | 0 | 0 |
| Whole-grain porridge | 87 | 305 | 100 | 174 | 150 | 261 |
| Rice and pasta[c] | 6 | 96 | 30 | 461 | 30 | 461 |
| Other grains | 9 | 124 | 19 | 296 | 35 | 545 |
| **Potatoes** | **14** | **46** | **30** | **102** | **25** | **85** |
| **Vegetables** | **71** | **163** | **188** | **269** | **152** | **186** |
| **Fruit, incl. juice** | **217** | **563** | **187** | **630** | **101** | **260** |
| Fruit and berries | 129 | 352 | 187 | 630 | 101 | 260 |
| Fruit smoothies | 58 | 156 | 0 | 0 | 0 | 0 |
| Fruit juice | 30 | 55 | 0 | 0 | 0 | 0 |
| **Legumes** | **6** | **28** | **15** | **166** | **38** | **553** |
| **Nuts** | **0** | **7** | **10** | **260** | **26** | **676** |
| **Added vegetable fats** | **12** | **274** | **15** | **407** | **20** | **733** |
| Vegetable oils | 1 | 35 | 5 | 183 | 20 | 733 |
| Margarine | 11 | 239 | 10 | 224 | 0 | 0 |
| **Dairy products** | **489** | **1495** | **260** | **619** | **128** | **262** |
| Milk | 357 | 655 | 160 | 247 | 80 | 124 |
| Yoghurt | 104 | 372 | 90 | 260 | 48 | 139 |
| Cheese | 21 | 268 | 10 | 112 | 0 | 0 |
| Butter | 7 | 200 | 0 | 0 | 0 | 0 |
| **Red meat** | **31** | **280** | **25** | **126** | **8** | **69** |
| Whole and minced red meat | 7 | 42 | 15 | 82 | 8 | 69 |
| Processed red meat | 24 | 238 | 10 | 43 | 0 | 0 |
| **Poultry** | **7** | **39** | **20** | **98** | **15** | **75** |
| Whole and minced poultry | 4 | 23 | 15 | 70 | 10 | 47 |
| Processed poultry | 3 | 16 | 5 | 28 | 5 | 28 |
| **Eggs** | **12** | **71** | **10** | **62** | **7** | **43** |
| **Fish** | **32** | **226** | **40** | **262** | **14** | **84** |
| Lean fish | 3 | 10 | 10 | 35 | 8 | 28 |
| Fatty fish | 8 | 76 | 20 | 188 | 6 | 56 |
| Processed fish products | 21 | 139 | 10 | 40 | 0 | 0 |
| **Sweets** | **89** | **330** | **30** | **229** | **30** | **229** |
| Sweets, cakes and desserts | 15 | 250 | 10 | 192 | 10 | 192 |
| Sugar-sweetened beverages | 42 | 80 | 20 | 37 | 20 | 37 |

| | | | | | | |
|---|---|---|---|---|---|---|
| Artificially sweetened beverages | 32 | 1 | 0 | 0 | 0 | 0 |
| **Drinking water** | **475** | **0** | **780** | **0** | **780** | **0** |
| **Other** | **29** | **82** | **0** | **0** | **0** | **0** |
| Plant-based beverages | 9 | 20 | | | | |
| Formula milk | 14 | 40 | | | | |
| Industrially produced toddler dinners | 5 | 15 | | | | |
| Added salt, condiments, etc. | 1 | 7 | | | | |

[a] Whole-grain content ≥50%
[b] Whole-grain content <50%
[c] Current diet includes whole-grain and refined rice and pasta. The scenario diets include whole-grain only.

**Supplementary Table S3** Content of macro- and micronutrients in the three EAT-*Lancet* scenario diets and the recommended intake for children aged 2-5 years according to the Norwegian nutrition recommendations [2]. The EAT-*Lancet* scenario follows the target values of the EAT-*Lancet* reference diet [3] adjusted for an energy intake of 5.3 MJ/d. The EAT-*Lancet* dairy and dairy & fish scenarios are based on the first scenario but includes double amounts of dairy products and dairy and fish products, respectively, still within the recommended intake ranges of the EAT-*Lancet* reference diet adjusted to 5.3 MJ/d

| | **EAT-*Lancet*** | **EAT-*Lancet* dairy** | **EAT-*Lancet* dairy & fish** | **Recommended intake** |
|---|---|---|---|---|
| Energy (MJ) | 5.3 | 5.3 | 5.3 | 5.3 |
| Protein (E%) | 16 | 18 | 19 | 10-20 |
| Total fat (E%) | 37 | 33 | 32 | 25-40 |
| *Saturated fat (E%)* | *6* | *7* | *7* | *<10* |
| *Monounsat. fat (E%)* | *17* | *14* | *14* | *10-20* |
| *Polyunsat. fat (E%)* | *10* | *9* | *8* | *5-10* |
| Total carbs. (E%) | 43 | 45 | 45 | 45-60 |
| *Added sugar (E%)* | *2* | *2* | *2* | *<10* |
| Dietary fibre (g/MJ) | 5 | 5 | 5 | >2-3 |
| Whole grain (g/MJ) | 22 | 22 | 22 | >7.5 |
| Vitamin A (µg) | 428 | 452 | 455 | 350 |
| Vitamin D (µg) | 1.3 | 1.6 | 2.4 | 10 |
| Vitamin E (mg) | 14 | 12 | 11 | 5 |
| Thiamine (mg) | 1.3 | 1.4 | 1.4 | 0.6 |
| Riboflavin (mg) | 0.8 | 1.0 | 1.0 | 0.7 |
| Niacin (mg) | 11 | 11 | 12 | 9 |
| B6 (mg) | 1.2 | 1.3 | 1.3 | 0.7 |
| Folate (µg) | 296 | 302 | 303 | 80 |
| B12 (µg) | 1.7 | 2.5 | 2.9 | 0.8 |
| Vitamin C (mg) | 59 | 59 | 59 | 30 |
| Calcium (mg) | 419 | 600 | 601 | 600 |
| Iron (mg) | 10.6 | 10.6 | 10.6 | 8 |
| Zinc (mg) | 7.5 | 8.1 | 8.1 | 6 |
| Iodine (µg) | 55 | 76 | 99 | 90 |
| Salt (g) | 1.6 | 1.8 | 1.8 | <3-4 |

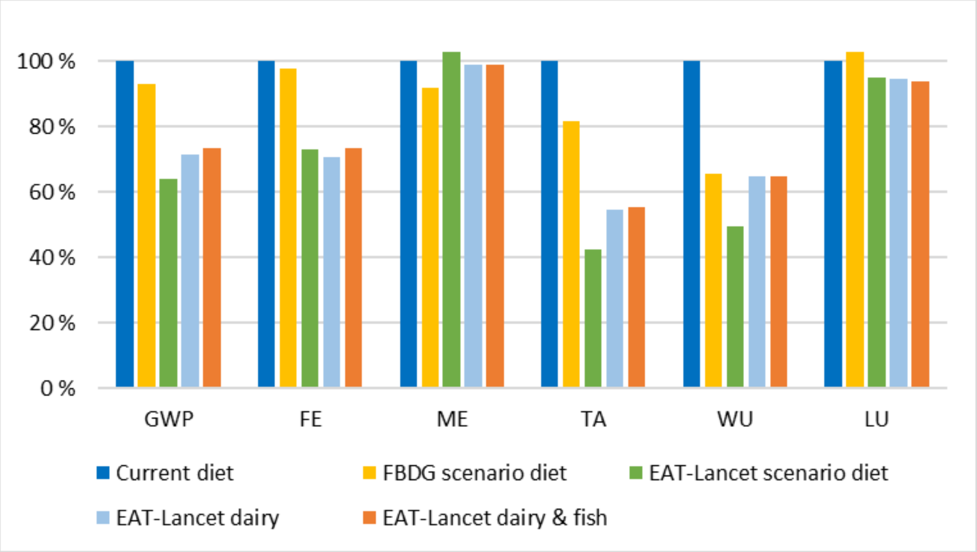


**Supplementary Fig. S1** Total environmental impact per impact category from the scenario diets in relation to the impacts from the current diet among 2-year-olds in Norway (Småbarnskost 3, *n* = 1413) [1]. FE, freshwater eutrophication; GWP, global warming potential; LU, land use; ME, marine eutrophication; TA, terrestrial acidification; WU, water use

## References:

1. Astrup, H., et al., *Småbarnskost 3. Landsomfattende undersøkelse av kostholdet blant 2-åringer i Norge [in Norwegian]*. 2020: Oslo.
2. Norwegian Directorate of Health, *Anbefalinger om kosthold, ernæring og fysisk aktivitet [in Norwegian]*. 2014: Oslo.
3. Willett, W., et al., *Food in the Anthropocene: the EAT-Lancet Commission on healthy diets from sustainable food systems.* Lancet, 2019. **393**(10170): p. 447-492.
